# Design of Compensation Coils for EMI Suppression in Magnetostrictive Linear Position Sensors

**DOI:** 10.3390/s120506395

**Published:** 2012-05-14

**Authors:** Yongjie Zhang, Weiwen Liu, Jinfeng Yang, Chunfeng Lv, Hui Zhao

**Affiliations:** School of Electronic, Information and Electrical Engineering, Shanghai Jiao Tong University, Shanghai 200240, China

**Keywords:** magnetostrictive linear position sensor, compensation coil, EMI suppression

## Abstract

This paper presents recent development on magnetostrictive linear position sensors (MLPS). A new compensation coil structure improves the EMI suppression and accuracy considerably. Furthermore, experimental results indicate that the new structure can improve the accuracy to ±0.13 mm nearly double the ±0.2 mm obtained with traditional structures. As another design continuation after the differential waveguide structure, this new structure is a practical and reliable implementation technique for the commercialization of MLPS.

## 1. Introduction

Linear position sensors are widely used for online measurement and control in industry [1–5]. The magnetostrictive linear position sensor (MLPS) based on the Wiedemann effect and Villari effect performs an accurate, long-range and non-contact position measurement. MLPS completes the measurement without contact between the cursor and the sensing rod; thus, the device can have a long service life and a high ingress protection level in harsh industrial conditions [6–8]. However, there are also some disadvantages in the use of MLPS due to the high-speed time measurement. The electromagnetic interference (EMI) or noise can lead to a great measurement error, so EMI suppression is the key design issue for MLPS [9–13].

Previous attempts have been made to improve accuracy of the sensor such as exploiting the interference of undamped echoes and controlling the excitation period Ferrari [14]. Hristoforou arranged two receiving coils at both ends of the sensor to obtain a better level of position sensitivity [15]. Zhang proposed a differential waveguide structure to get a higher accuracy of MLPS [16]. This compensation coil structure is a continuation of Zhang's research.

When MLPS is applied to the fluid cylinder and piston cylinder, the available space in the measurement direction is quite narrow. Therefore we need some improvement of the sensor structure to enhance the EMI suppression without adding additional size. In the present paper, we illustrate a compensation coil that can improve the EMI suppression and accuracy of the sensor. The proposed structure has been patented to reserve the authors' rights on the use of the device.

## 2. Principle of the MLPS

**Figure 1.**
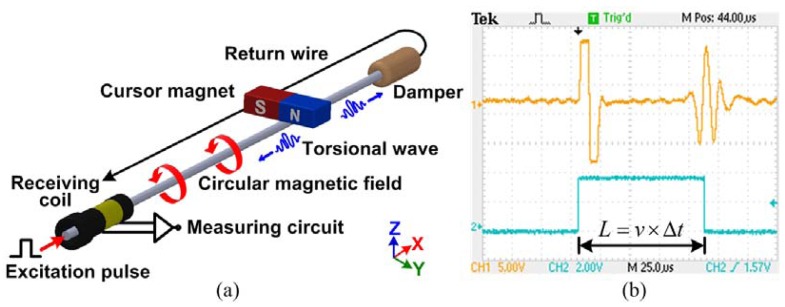
MLPS principle. (**a**) MLPS operation; (**b**) Oscilloscope waveform of the induction signal [16].

The principle of MLPS is illustrated in Figure 1a. The emitter on the measurement circuit periodically generates an excitation pulse through the ferromagnetic material waveguide, causing a circular magnetic field around. The interaction between the magnetic fields of the cursor magnet and excitation pulse produces a rotation of the magnetic domains in the waveguide.

According to the Wiedemenn effect, two torsional waves are created in the waveguide in both directions away from the position of the cursor magnet at a certain speed. The coil at one end of the waveguide is the receiver of the sensor while the other end connects to the damper. The damper absorbs the torsional wave to avoid it from reflecting back and corrupting the data at the other end. When the torsional wave arrives at the receiving coil, the flux lines of the residual magnetic field change. As described by the Faraday-Lenz Law, the change in permeability induces a voltage variation at the receiving coil output. The position of the cursor magnet can then be computed from the propagation time as the torsional wave travels from the cursor magnet to the receiving coil [17–20]. An example of the oscilloscope waveform at the receiving coil is shown in Figure 1(b). If we get the propagation time _Δ_*_t_*, with the knowledge of the propagation speed *v*, the position *L* is computed as:

(1) L=v×Δt

Because of the electromagnetic effects in the sensor, MLPS is susceptible to EMI. The instability of the induction signal will cause random errors in high-speed time measurement due to EMI [21,22]. Unfortunately, the random error caused by EMI, which cannot be compensated in the calibration process, is the major cause of the measurement uncertainty. To improve the EMI suppression and accuracy of the sensor radically, we propose a new MLPS structure with a compensation coil, which will be discussed in the next section.

## 3. Compensation Coil Structure of the MLPS

**Figure 2.**
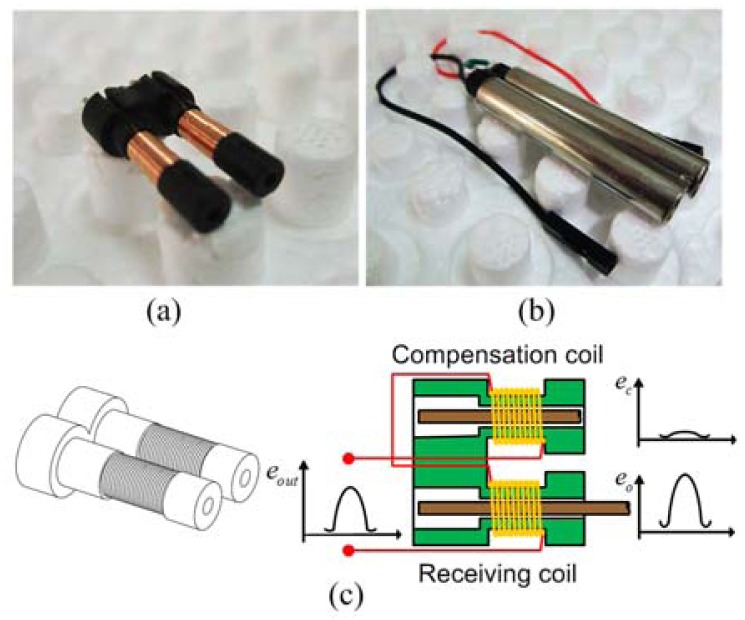
Structure of compensation coil (**a**) Receiving coil with compensation coil; (**b**) Coils installed with screening sleevings and connecting wires; (**c**) Schematic diagram of the compensation coil.

The compensation coil structure of the magnetostrictive linear position sensor is explained in Figure 2. In Figure 2(a), the parameters of compensation coil and receiving coil are exactly the same, including the number of turns, length, wire diameter, inner diameter and so on. As shown in Figure 2(b), when the sensor works, the compensation coil and receiver coil are installed with two same parameter screening sleevings. The screening sleevings which are made of Ni-Fe alloy have good shielding effectiveness. Furthermore, two sleevings contact each other to ensure two coils induce same intensity EMI at the same time. Figure 2(c) is the schematic diagram of the compensation coil. In order to achieve the best compensation effect, we place a waveguide in the compensation coil as same as in the receiving coil. Besides, the compensation coil and receiving coil are connected in a head-to-tail position at the measurement circuit in order to form a differential structure. Therefore, the EMI effects of the new structure sensor will be neutralized. Consequently, the new sensor with compensation coil has higher EMI suppression than the traditional structure sensor in theory.

The compensation coil is close to the receiving coil. This simple improvement will not increase the size and complexity of the sensor, which is easy to apply in the industrialization of the MLPS. As we known, most of the EMI of MLPS is common-mode interference. Besides when the receiving coil and compensation coil are connected with a differential structure, the output of the compensation coil, which is caused by the EMI, will reduce the EMI interference according to the sensor model [23,24]. In other words, a MLPS with a compensation coil has a high EMI suppression, which will increase the accuracy of the sensor. In Zhang's paper, a sensor model was proposed, which we can use to discuss the compensation coil based on [16]:

(2) $$e_{0}=-NS\frac{HvM_{s}^{2}k^{2}}{2L_{0}\left(KAk^{2}+6\pi \mu \lambda^{4}\right)}$$

where *e*_0_ is the induced voltage, *N* is the turns of the receiving coil, *S* is the cross-sectional area of the receiving coil, *H* is the magnet field, *v* is the velocity of the torsional wave, *M_s_* is the saturation magnetization, *k* is the electromechanical coupling coefficient, *L*_0_ is the length of the receiving coil, *K* is the magnetic anisotropy constant, *A* is the cross-sectional area of the waveguide, *μ* is the reversible permeability, and *λ* is the magnetostrictive constant.

The induction signal of induction coil is written as:

(3) $$e_{0}=s_{0}+n_{0}$$

where *s*_0_ is caused by the torsional wave, *n*_0_ is caused by the noise, like electromagnetic interference, power ripples, and vibration noise.

The induction signal of compensation coil is written as:

(4) $$e_{1}=n_{1}$$

Besides, the noise which is received by the induction coil and compensation coil is the same, *n*_0_ = *n*_1_. The output of the sensor can be rewritten as:

(5) $$e=e_{0}-e_{1}=s_{0}$$

From the above expression, the compensation coil can reduce the influence of electromagnetic noise and increase the signal to noise ratio of the MLPS.

## 4. Results

**Figure 3.**
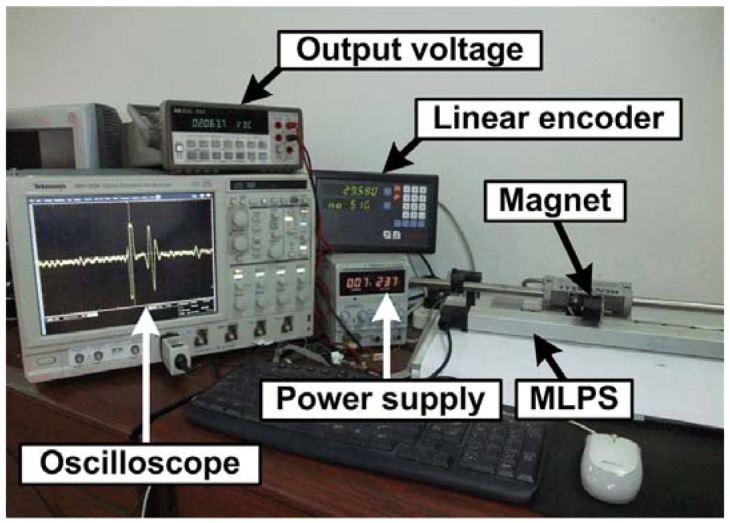
Experimental setup.

The MLPS experimental setup is shown in Figure 3. The sensor is fixed to an immobile bench which also holds a rail with a moving tower.

The cursor magnet is attached to the moving part and can move above the sensor only in measurement direction. The position of the cursor magnet is measured by means of a linear encoder with the resolution of 5 μm through the experiment, the vertical distance between cursor magnet and waveguide remains invariant.

In order to compare the performance of the compensation coil, we made two magnetostrictive linear position sensors with 300 mm measurement range. One has a compensation coil while the other doesn't. The parameters of the compensation coil are exactly the same as the receiving coil. Furthermore, the experimental conditions are exactly same, as listed in Table 1.

**Figure 4.**
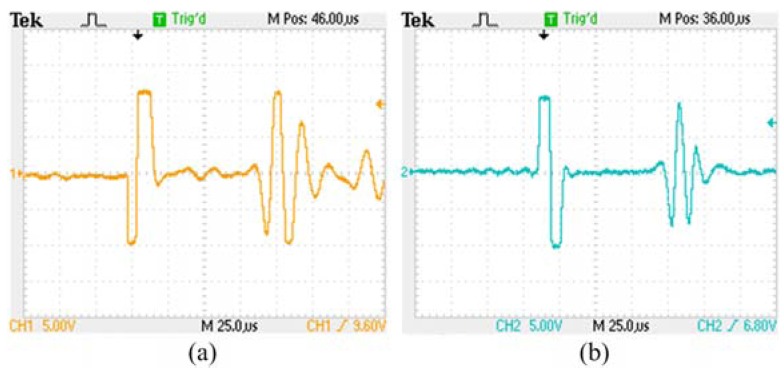
Oscilloscope waveforms of induction signal (**a**) without the compensation coil; (**b**) with the compensation coil.

Figure 4(a,b) show the oscilloscope waveforms of induction signal without the compensation coil and with the compensation coil, respectively. Without the compensation coil, we can get a higher signal intensity, but more signal burrs. The signal burrs will cause a loss of accuracy of the sensor. When the compensation coil is installed in the MLPS, the signal burrs are noticeably decreased. With this result, we can obtain an ideal induction signal with higher EMI suppression with the compensation coil. Therefore, the compensation coil reduces the influence by EMI.

Signal to noise ratio and accuracy are important indexes of magnetostrictive linear position sensors. In the actual research, we found they are all subject to the influence of the induction signal quality. When induction signal jitter is reduced, the signal-to-noise ratio and accuracy of the sensor get higher. Furthermore after the calibration, nonlinearity of the MLPS is determined by the induction signal instability, which means the jitter range of the induction signal on the timeline when the cursor magnet maintains a constant position. Therefore we can quantify the signal-to-noise ratio with the induction signal instability on the other side.

**Figure 5.**
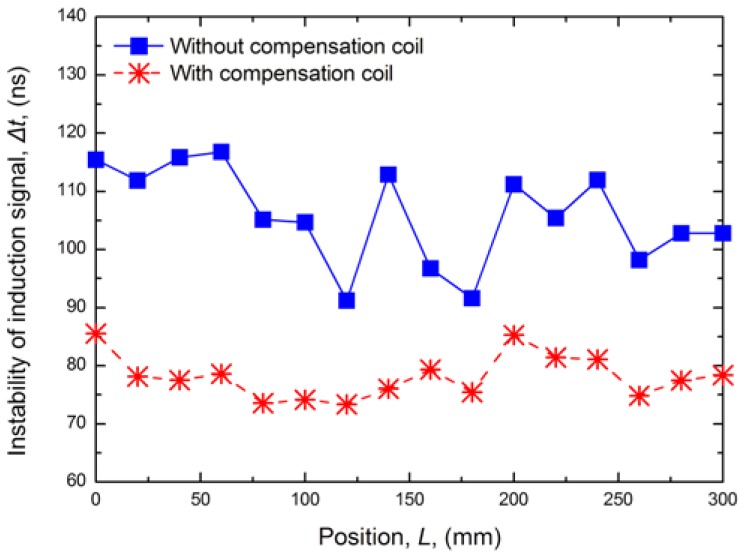
Comparison of induction signal instability.

The induction signal instability means the random jitter of the induction signal on the timeline from the standpoint of observation. The signal instability can be directly observed on an oscilloscope. When we measure the induction signal instability, the position magnet and the sensor are fixed, and we collect 1,000 sets of data continuously within one hour with a high-speed oscilloscope (Tektronix DPO 7254). We assume that the induction signal jitter is consistent with the normal distribution, and we can calculate the induction signal instability by compiling the variation of induction signal on the reference voltage value. (σ = 2, Probability = 95%). With the compensation coil, the induction signal instability calculated by MCU is reduced from 100 to 80 ns, as shown in Figure 5.

**Figure 6.**
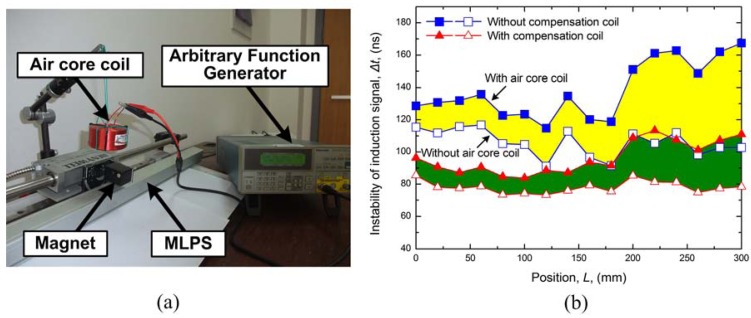
MLPS EMI suppression experiment with the air core coil (**a**) Experimental setup; (**b**) Comparison of induction signal instability with the perturbation.

In a further evaluation by EMI suppression, we set an air core coil above the sensor and cursor magnet. An excitation signal consisting of a settled sine train created by an arbitrary function generator (Tektronix AFG310) flows through the air core coil as shown in Figure 6(a). This movement can guarantee that the EMI induced by the air core coil is invariant. When the air core coil is halfway through the measurement range, we move the position magnet to complete the full scale measurement and get the induction signal instability curve as shown in Figure 6(b). It can be seen from the figure that when the air core coil approaches the receiving coil, a greater perturbation is induced. Furthermore when the interferential coil is near or after the position magnet in measurement direction, less perturbation will be induced due to the magnetic field interaction and damping. The increment of instability with compensation coil is smaller than the sensor without compensation coil. Therefore, a higher EMI suppression could be obtained with the compensation coil.

**Figure 7.**
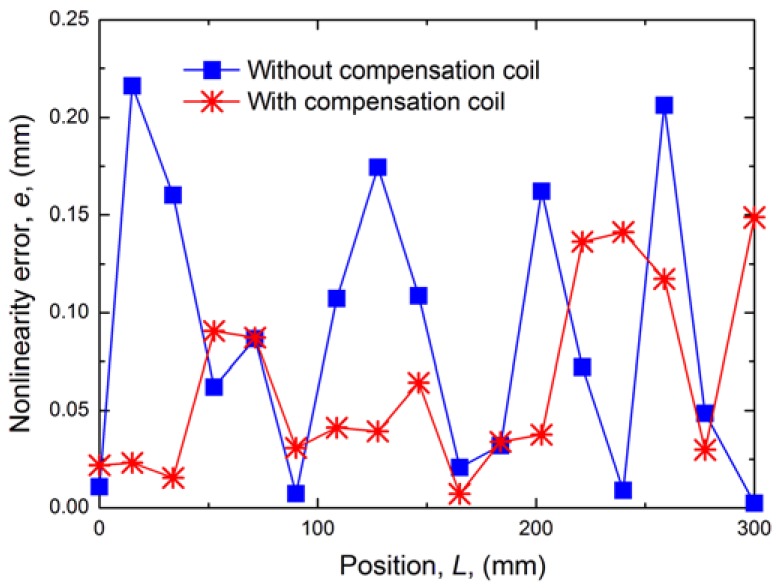
Comparison of linear error.

The absolute value of the linear error is shown in Figure 7. The mean deviation over all positions with compensation coil is reduced from 0.0875 mm to 0.0581 mm. So the experimental results indicate that the compensation coil can improve accuracy to ±0.13 mm higher than ±0.2 mm without the compensation coil. Apparently, a higher accuracy will be obtained with higher EMI suppression.

## 5. Conclusions

In the present paper, we have proposed a new magnetostrictive linear position sensor structure with a compensation coil. The new sensor provides higher EMI suppression and linear precision. The present study provides a practical and reliable implementation technique for the commercialization of the MLPS.

## Figures and Tables

**Table 1.** Experimental conditions for the MLPS.

**Item**	**Parameter**	**Symbol [Unit]**	**Material, Value**
Magnetostrictive wire	Material		Ni-Span-C
	Length	L_s_ [mm]	300
	Diameter of wire	d [mm]	0.5
	Torsional modulus	E [kN mm^−2^]	70
	Resistance	R [Ω]	3.6

Pulse current	Amplitude	I_p_ [A]	3
	Period	T_w_ [ms]	1
	Width of pulse	T_p_ [μs]	5

Cursor magnet	Material		Ceramic
	Size	[mm]	10 × 30 × 8.5

Amp.	Instrument Amplifers	[Av dB]	49.5 + 20

Comparator	Comparison voltage	Vref [V]	3.6

Receiving coil and	Number of turns	N [turn]	800
Compensation coil	Length	L_c_ [mm]	5.2
	Wire diameter	d_w_ [mm]	0.05

Torsional wave	Speed	v [m s^−1^]	2650
